# Short‐term outcomes of patients undergoing endoscopic submucosal dissection for colorectal lesions

**DOI:** 10.1002/deo2.136

**Published:** 2022-06-16

**Authors:** Yuki Nakajima, Daiki Nemoto, Tetsutaro Nemoto, Yosuke Takahata, Masato Aizawa, Kenichi Utano, Noriyuki Isohata, Shungo Endo, Alan Kawarai Lefor, Kazutomo Togashi

**Affiliations:** ^1^ Department of Coloproctology, Aizu Medical Center Fukushima Medical University Fukushima Japan; ^2^ Department of Surgery Jichi Medical University Tochigi Japan

**Keywords:** colorectal neoplasm, endoscopic submucosal dissection, length of stay, perioperative complications, short‐term outcomes

## Abstract

**Objectives:**

Endoscopic submucosal dissection (ESD) of colorectal lesions was invented in Japan, but postoperative management including hospital stay has not been reconsidered due to the Japanese insurance system. To explore appropriate postoperative management after colorectal ESD, we reviewed short‐term outcomes after ESD in non‐selected consecutive patients.

**Methods:**

Patients who underwent colorectal ESD from April 2013 to September 2020 in one institution were reviewed. The primary outcome measure was the occurrence of adverse events stratified by the Clavien‐Dindo classification with five grades. A logistic regression model with the Firth procedure was applied to investigate predictors of severe (grade III or greater) adverse events.

**Results:**

A total of 330 patients (female 40%, male 60%; median 72 years; IQR 65–80 years) with colorectal lesions (median 30 mm, IQR 23–40 mm; colon 77%, rectum 23%; serrated lesion 4%, adenoma 47%, mucosal cancer 30%, invasive cancer 18%) was evaluated. The en bloc resection rate was 97%. The median dissection time was 58 min (IQR: 38–86). Intraprocedural perforation occurred in 3%, all successfully treated by endoscopic clipping. No delayed perforations occurred. Postprocedural bleeding occurred in 3% on days 1–10 (median day 2); all were controlled endoscopically. Severe adverse events included only delayed bleeding. In analyzing severe adverse events in a multivariate logistic regression model with the Firth procedure, antithrombotic agent use (*p* = 0.016) and rectal lesions (*p* = 0.0010) were both significant predictors.

**Conclusions:**

No serious adverse events occurred in this series. Four days of hospitalization may be too long for the majority of patients after ESD.

## INTRODUCTION

Colorectal endoscopic submucosal dissection (ESD) is an effective treatment that enables en bloc resection of large lesions with a lower recurrence rate compared with conventional endoscopic resection.^1^, ^2^ The procedure has a risk for the development of adverse events including periprocedural perforation and bleeding,^3^, ^4^, ^5^ but the occurrence of these events is decreasing with advances in procedural strategy and endosurgical devices.^6^, ^7^, ^8^, ^9^ For instance, an obvious or imminent perforation or bleeding can be adequately treated endoscopically by the placement of clips.^10^

However, the policy for the management of post‐ESD patients has not changed significantly for about 20 years since the ESD technique was introduced. Patients who undergo colorectal ESD are typically hospitalized for a few (2–5) days after the procedure in Japan. Although ESD was invented in Japan, postoperative management including hospital stay has not been reconsidered due to the Japanese single‐payer national health insurance system in which provider reimbursement is calculated based on a flat‐rate per‐diem fee based on the diagnosis group.^11^ The current situation in the United States was reported by Draganov et al, where 70% of post‐ESD patients undergo outpatient care,^12^ albeit possessing a selection bias based on ambiguous treatment criteria for ESD. Therefore, their management strategy may not be adaptable to ESD procedures performed around the world.

The coronavirus disease 2019 (COVID‐19) pandemic resulted in major changes in overall health care strategy.^13^, ^14^, ^15^ Hospitalization has to be restricted during COVID‐19 epidemic peaks. In this situation, unnecessary hospitalizations are minimized after ESD. In 2017 before the COVID‐19 crisis, Ohya et al investigated the feasibility of colorectal ESD as an outpatient procedure in a prospective setting and reported that 91% (156/171) of post‐ESD patients were discharged 2–4 h later after completion of ESD.^16^ In 2021, Pecere et al reported that day surgery was feasible for 13 rectal ESD and two colonic ESD procedures.^17^ In these two reports, however, patients were highly selected and not consecutive. In other words, the indication for early discharge was limited to facile ESD procedures performed in low‐risk patients.

To explore the appropriate postoperative management and feasibility of shortening the hospital stay after colorectal ESD, this study reviewed the current short‐term outcomes of ESD in non‐selected consecutive patients.

## METHODS

### Study design

We conducted a single‐center, observational study. This study was reviewed and approved by the Institutional Review Board of Fukushima Medical University (registration No. 2020‐255) in accordance with the Declaration of Helsinki. All patients or their families provided signed informed consent. All data were collected by November 2020. The Strengthening the Reporting of Observational Studies in Epidemiology (STROBE) Statement was followed in reporting this study.

### Patients

We reviewed consecutive patients who underwent colorectal ESD at the Aizu Medical Center Fukushima Medical University from March 2013 to September 2020. Patients who underwent ESD for multiple lesions on the same day were excluded, but those who underwent multiple ESD on different days with a ≥1‐month interval were included. The indications for colorectal ESD followed the Colorectal ESD Standardization Implementation Working Group and Colorectal ESD/EMR Guidelines established by the Japan Gastroenterological Endoscopy Society.^2^ Patients taking antithrombotic agents were treated according to the Japanese guideline.^18^, ^19^, ^20^

### ESD procedure

The ESD procedure was performed using an EC‐590MP (Fujifilm, Tokyo, Japan) with a DH‐29CR (Fujifilm) at the tip of the colonoscope or an EC‐L600ZP/ZP7 (Fujifilm) with DH‐34CR (Fujifilm) and carbon dioxide insufflation. Needle knives specially designed for ESD with minor modifications to the diathermy tip (Dual knife, KD‐650Q; Olympus, Tokyo, Japan or 1.5‐mm FlushKnife‐BTs, DK2620JBS, Fujifilm) were used for the ESD procedure with a high‐frequency generator (ERBE, Elektromed‐VIO300D; Tubingen, Germany). All procedures were performed under the supervision of a single expert endoscopist (Daiki Nemoto).

### Patient management after ESD

**FIGURE 1:**
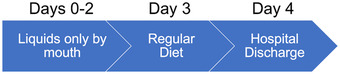
Clinical pathway

After ESD, nursing staff and attending physicians carefully monitored the patients with special attention to the onset of fever, abdominal pain, and lower gastrointestinal bleeding. To detect post ESD electrocoagulation syndrome or delayed perforation, patients manifesting fever or abdominal pain underwent an abdominal CT scan at the discretion of the attending physicians. Patients completing ESD without the occurrence of adverse events were permitted to drink water immediately after ESD but were otherwise fasted and received intravenous fluids for 2 days. Solid food ingestion was started on day 3, followed by discharge on day 4 (Figure 1). The management followed an existing clinical pathway.

### Outcome measurements

The primary outcome of this study was the occurrence of postoperative adverse events, which were classified into five grades according to the Clavien–Dindo classification.^21^ This classification system is well known and ranks adverse events objectively and reproducibly. As shown in Table S1, grade I events include any deviation from the normal course that does not require pharmacological treatment or surgical, endoscopic intervention. These events require therapy limited to antiemetics, antipyretics, analgesics, and diuretics. Grade II events include those requiring, pharmacological treatment with drugs other than those allowed for grade I as well as blood transfusions or antibiotic agents. Grade III events require a surgical, endoscopic, or radiologic intervention. Grade IV events are life‐threatening adverse events involving single organs or multiorgan dysfunction. Death is classified as a Grade V event. Patients with no adverse events were defined as grade 0. Fever was defined as ≥37.5°C, considering post ESD electrocoagulation syndrome.^22^ Abdominal pain included mild tenderness and subjective spontaneous pain.

Secondary outcomes were defined descriptively including patient demographics, lesion characteristics (location, size, morphology, histology, and depth of invasion), en bloc resection rate, interrupted resection rate, intra‐procedural perforation, cutting time, clinical course after ESD, blood tests (white blood cell count, C‐reactive protein) on day 1. The location of the lesions was classified into the proximal colon (from cecum to transverse colon), distal colon (descending and sigmoid colon), and rectum. In analyzing cutting time, interrupted cases were excluded. The morphology was evaluated according to the Paris classification,^23^ and “0‐Is and 0‐Is+IIc” were classified into polypoid type whereas “0‐IIc, 0‐IIa” and “0‐IIa+IIc” were classified as the flat type. Histology was classified as adenoma, cancer, sessile serrated lesion (SSL), or others. Cancer was subcategorized by the depth of invasion following the Japanese Classification of Colorectal, Appendiceal, and Anal Carcinoma.^24^ In this system, Tis represents cancer in situ (mucosal cancer), T1a represents cancer invading the submucosa less than 1 mm, and T1b represents cancer invading the submucosa more than 1 mm.

### Statistical analysis

All analyses were exploratorily performed, thus sample size was not calculated prior to commencement of the study. Data are presented as the median (interquartile range [IQR]) or true numbers if appropriate. The chi‐squared test was used for nominal data, and the Mann–Whitney *U*‐test was used for quantitative data. All *p*‐values are two‐tailed. *p*‐values <0.05 are considered to indicate statistical significance. We evaluated predictive factors for the development of severe adverse events (Clavien‐Dindo grade III/IV/V) using a logistic regression model with the Firth procedure because severe adverse events were rare.^25^, ^26^ The Firth procedure provides bias‐reduction for small size as well as yielding finite and consistent estimates even in case of separation. All statistical analyses were performed with Stata 16 (Stata Corp., College Station, Texas, USA).

## RESULTS

### Background characteristics (Table 1)

**FIGURE 2:**
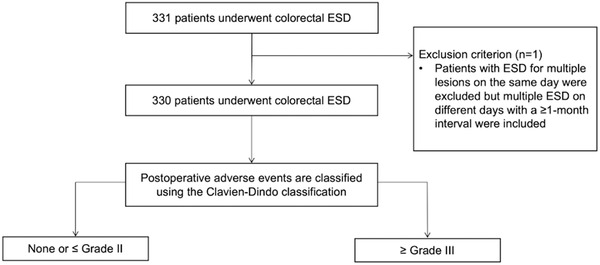
Study flow chart

A total of 331 patients underwent ESD at the Aizu Medical Center at Fukushima Medical University from March 2013 to September 2020. One patient was excluded due to multiple lesions treated in one patient on the same day. Finally, 330 patients were included in the study (Figure 2). The characteristics of patients, lesions, and procedures are summarized in Table 1. One (0.3%) procedure was interrupted due to severe fibrosis in the submucosal layer, and surgical resection was performed one month later. Intra‐procedural perforation occurred in 10 (3.3%) patients, all treated by clipping. The median cutting time was 58 minutes (IQR 38–86 min).

**TABLE 1:** Background characteristics

**Patients**	
Number of patients	330
Age (years), median (IQR)	72 (65–80)
Male gender, *n* (%)	198 (60.0)
Female gender, *n* (%)	132 (40.0)
Antithrombotic agent use, *n* (%)	50 (15.2)
**Lesion**	
Number of lesions	330
Location, *n* (%)	
Proximal colon	198 (60.0)
Distal colon	56 (17.0)
Rectum	76 (23.0)
Size (mm), median (IQR)	30 (23–40)
Morphology, *n* (%)	
Flat	152 (46.1)
Polypoid	176 (53.3)
Other	2 (0.6)
Histology, *n* (%)	
Adenoma	156 (47.3)
Tis (mucosal cancer)	98 (29.7)
T1a (submucosal invasion <1 mm)	27 (8.2)
T1b (submucosal invasion ≥1 mm)	34 (10.3)
SSL	12 (3.6)
Others^†^	3 (0.9)
**Procedure**	
en bloc resection, *n* (%)	320 (97)
Interrupted resection, *n* (%)	1 (0.3)
Intra‐procedural perforation, *n* (%)	10 (3.3)
Cutting time (min), median (IQR)	58 (38–86)

IQR: interquartile range; SSL: sessile serrated lesion.

† Others include one mucosal prolapse syndrome, one no residual tumor after previous endoscopic resection, and one dysplasia‐associated ulcerative colitis.

**TABLE 2:** Clinical course after endoscopic submucosal dissection (ESD)

	**All (*n* = 330)**
Laboratory tests on day 1^†^	
WBC (/μl), median (IQR)	7500 (6100–8930)
CRP (mg/dl), median (IQR)	0.45 (0.20–1.08)
Postoperative adverse events,^‡^ *n* (%)	
Grade 0	279 (84.5)
Grade I	11 (3.3)
Grade II	31 (9.4)^§^
Grade III	9 (2.7)^¶^
Grade IV/V	0 (0)
Hospital stay	
Length of hospital stay (days), median (IQR)	4 (4–4)

ESD: endoscopic submucosal dissection; IQR: interquartile range; WBC: white blood cell count; CRP: C‐reactive protein

^†^ 26 WBC and 29 CRP levels are lacking.

^‡^ Postoperative adverse events were evaluated using the Clavien‐Dindo classification.

^§^ All were treated with antibiotic administration.

^¶^ All were episodes of delayed bleeding successfully treated endoscopically.

**TABLE 3:** Severity of each postoperative adverse event by Clavien‐Dindo classification

**Adverse events**	**Fever (*n* = 20)**	**Abdominal pain (*n* = 30)**	**Delayed bleeding (*n* = 9)**	**Delayed perforation (*n* = 0)**
Grade I, *n* (%)	5 (25)	6 (20)	0 (0)	0 (0)
Grade II, *n* (%)	15^†^ (75)	24^†^ (80)	0 (0)	0 (0)
Grade III, *n* (%)	0 (0)	0 (0)	9 (100)	0 (0)
Grade IV/V, *n* (%)	0 (0)	0 (0)	0 (0)	0 (0)

^†^ 10 overlapping.

**TABLE 4:** Features of patients with Clavien‐Dindo classification grade ≥ III adverse events

**Factor**	**Grade ≤ II (*n* = 321)**	**Grade ≥ III (*n* = 9)**	***p*‐value**
Age (years), median (IQR)	72 (65–80)	62 (60–65)	0.024*
Gender, *n* (%)			
Male	190 (59.2)	8 (88.9)	0.073**
Female	131 (40.8)	1 (11.1)	
Antithrombotic agent use, *n* (%)	46 (14.3)	4 (44.4)	0.013**
Lesion location, *n* (%)			
Proximal colon	196 (61.0)	2 (22.2)	<0.0001**
Distal colon	56 (17.5)	0 (0)	
Rectum	69 (21.5)	7 (77.8)	
Lesion size (mm), median (IQR)	30 (23–40)	31 (20–46)	0.062*
Morphology, *n* (%)			
Flat	151 (47.0)	3 (33.3)	0.42**
Polypoid	170 (53.0)	6 (66.7)	
Histology, *n* (%)			
Adenoma	152 (47.4)	4 (44.4)	0.72**
Tis (mucosal cancer)	96 (29.1)	2 (22.2)	
T1a (submucosal invasion <1000μm)	25 (7.8)	2 (22.2)	
T1b (submucosal invasion ≥1000μm)	33 (10.3)	1 (11.1)	
SSL	12 (3.7)	0 (0)	
Others^†^	3 (0.9)	0 (0)	
Cutting time (min), median (IQR)	56 (38–81)	72^‡^ (63–91)	0.22*
Intra‐procedural perforation, *n* (%)	10 (3.1)	0 (0)	0.59**

IQR: interquartile range; SSL: sessile serrated lesion.

Postoperative adverse events were evaluated using the modified Clavien‐Dindo grade and categorized into two groups; ≤ grade II and ≥ grade III.

^†^ Others include 1 mucosal prolapse syndrome, 1 no residual tumor after previous endoscopic resection, and 1 dysplasia associated ulcerative colitis.

^‡^ One interrupted case is excluded.

* Mann–Whitney *U*‐test.

** Chi‐squared test.

**TABLE 5:** Predictors of Clavien‐Dindo classification grade ≥ III adverse events

	**Univariate analysis** **^†^**		**Multivariate analysis** **^†^**	
**Factor**	**OR (95% CI)**	***p*‐value**	**OR (95% CI)**	***p*‐value**
Age (years)				
<75	1	0.31		
≥75	0.47 (0.11–2.0)			
Gender				
Female	1	0.13		
Male	3.9 (0.68–22)			
Antithrombotic agent use				
Absent	1	0.016	1	0.030
Present	4.8 (1.3–17)		4.4 (1.2–17)	
Lesion location				
Colon	1	0.0010	1	0.0020
Rectum	10 (2.5–46)		10 (2.4–44)	
Lesion size (mm)				
<50	1	0.45		
≥50	1.8 (0.41–7.6)			
Morphology				
Polypoid	1	0.46		
Flat	0.61 (0.16–2.3)			
Histology				
Non‐T1b	1	0.64		
T1b	1.5 (0.26–8.9)			
Cutting time^‡^ (min)				
<120	1	0.70		
≥120	1.4 (0.24–8.3)			
Intra‐procedural perforation				
Absent	1	0.76		
Present	1.6 (0.085–29)			

CI: confidence interval; OR: odds ratio.

^†^ Logistic regression model with the Firth procedure.

^‡^ One interrupted endoscopic submucosal dissection is excluded from the analysis.

### Clinical course after ESD (Tables 2 and 3, and Figure 3)

The vast majority of patients had no remarkable abnormalities in blood tests on post‐procedure day 1 (white blood cell count (WBC: median 7500/μl, maximum 16,200/μl; C‐reactive protein: median 0.45 mg/dl, maximum 14.8 mg/dl). Postprocedural adverse events did not occur in 279 (84.5%) patients but adverse events classified as Clavien‐Dindo grade ≥ I occurred in the remaining 51 patients (15.5%). Grade II adverse events included fever and/or abdominal pain treated non‐operatively with the administration of intravenous antibiotics, and all grade III adverse events included delayed bleeding successfully treated with endoscopic clipping. No patient developed a grade ≥ IV adverse event including delayed perforation. The median hospital stay was 4 days (IQR 4–4) as scheduled in advance.

**FIGURE 3:**
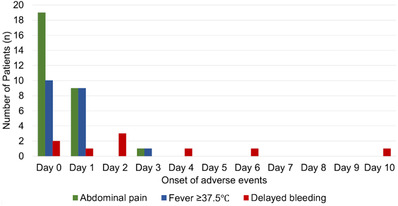
Onset of adverse events after endoscopic submucosal dissection (ESD)

The development of adverse events after ESD is summarized in Figure 3. Six of nine episodes of delayed bleeding occurred from days 0 to 2, and the remaining three, who received antithrombotic therapy and resumed it on day 2, bled on days 4, 6, and 10. All delayed bleeding patients were successfully treated endoscopically without a decrease in serum hemoglobin level ≥ 2 g/dl but five patients had extended hospital stays. Twenty (6.0%) patients manifested a fever on days 0 or 1, and 15 patients were classified as grade II. Thirty (9.1%) patients reported abdominal pain on days 0 or 1, and 12 patients were classified as grade II. Except for one patient, fever and abdominal pain occurred on days 0 or 1. The exceptional patient who underwent ESD for a 51 mm lesion in the ascending colon developed abdominal pain and fever on day 3 after starting oral intake, being treated non‐operatively with antibiotics, and was discharged on day 5.

### Features of patients with Clavien‐Dindo classification grade ≥ III adverse events (Table 4)

All Clavien‐Dindo classified grade III adverse events had delayed bleeding. There were no significant differences in gender, lesion size, morphology, histology, cutting time, or intraprocedural perforation. In contrast, the age of patients with grade ≥ III adverse events was lower than that of patients with lower‐grade adverse events (*p* = 0.024). The rate of antithrombotic agent use was higher in a patient with grade ≥ III adverse events compared with lower grade events (*p* = 0.013). Patients with rectal lesions had a higher incidence of grade III adverse events (*p* < 0.0001). The features of patients with grade ≥II adverse events are shown in Table S2.

### Predictors of Clavien‐Dindo classification grade III adverse events (Table 5)

In a logistic regression model with the Firth procedure, univariate analysis showed that antithrombotic agents use (*p* = 0.016) and lesion location (*p* = 0.0010) are significantly associated with grade ≥ III adverse events. In multivariate analysis, using two covariates with *p* < 0.1 in univariate analysis, antithrombotic agents use (*p* = 0.030) and lesion location (*p* = 0.0020) were still significant predictors for adverse events of grade ≥ III. Analysis of predictors for grade ≥ II adverse events is shown in Table S3.

## DISCUSSION

This study reviews the short‐term outcomes of non‐selected consecutive patients after colorectal ESD. Severe (Clavien‐Dindo classification grade ≥ III) postoperative adverse events included only delayed bleeding which was successfully treated with endoscopic clipping. All patients with postoperative fever and abdominal pain were also treated non‐operatively with 75% of these patients receiving intravenous antibiotics.

The strength of the present study is the examination of non‐selected consecutive ESD patients which represent a real‐world colorectal ESD patient cohort in an academic medical center. Another strength of this study is that it includes a relatively recent series of patients who underwent colorectal ESD. In 2015, Tomiki et al. reported a clinical pathway to discharge patients 3 days after colorectal ESD, although short‐term outcomes were not excellent in their retrospective analysis.^27^ In contrast, the present series has a higher en bloc resection rate and a shorter resection time. A subsequently lower rate of adverse events was achieved compared with previous reports.^1^ Accordingly, the short‐term outcomes in the present series imply that shortening the hospital stay after ESD may be feasible in most patients at this time.

In surgery, the severity of postoperative adverse events is classified using the Clavien‐Dindo classification. In endoscopy, however, a widely used classification is the American Society for Gastrointestinal Endoscopy consensus criteria.^28^ In the American Society for Gastrointestinal Endoscopy criteria, a prolonged hospital stay was used as an indicator of the severity of postoperative adverse events. In Japan, however, the American Society for Gastrointestinal Endoscopy criteria do not appropriately reflect the occurrence of adverse events because the length of hospital stay is based on a flat‐rate per‐diem fee based on the diagnosis group.^11^ In the present study, we applied the Clavien‐Dindo classification to assess the severity of postoperative adverse events, although the patients receiving a blood transfusion could be classified as grade II.

In the present series, the overwhelming majority of patients with postprocedural fever and abdominal pain developed these problems on day 0 or day 1. Accordingly, clinical assessment on day 1 could predict the hospital length of stay for most patients. All patients who manifested fever and abdominal pain were treated non‐operatively in this series, although a few patients delayed the start of oral intake all started clear liquids on schedule. Hospital stay could be shortened by changing intravenous antibiotics to oral administration or stopping this for those patients who needed antibiotics. This management strategy may be reasonable because the latest evidence demonstrates that prophylactic administration of antibiotics is not necessary.^29^

The use of antithrombotic agents and the presence of rectal lesions were both predictors for developing severe adverse events (grade ≥ III) which were all delayed bleeding. In previous studies,^30^, ^31^, ^32^ these two factors were also identified as risk factors for the development of delayed bleeding after colorectal ESD. In our most recent patients who underwent ESD, therefore, hemostasis is normally achieved for visible vessels in the wound, particularly in rectal lesions. Since most patients with delayed bleeding developed it from day 0 to day 2 in the present series, a two‐day hospital stay would be sufficient in usual clinical practice. Conversely, patients with predictors for the development of delayed bleeding should be observed as inpatients.

In clinical practice, a 1 or 2‐day stay for ESD gives patients clinical benefits. First, a shorter hospital stay results in reduced medical costs. Second, patient accessibility to ESD is facilitated, even with the number of inpatient beds due to the COVID‐19 pandemic. Some patients with early‐stage colorectal cancer postponed their treatment because of the pandemic.^33^ Given that a shorter hospital stay is useful in practice, more patients will be able to undergo colorectal ESD without delay.

This study has several acknowledged limitations. First, this is a retrospective observational study conducted in a single center. There was no attempt to shorten the hospital stay after colorectal ESD for patients in this study. The feasibility of shortening the hospital stay after colorectal ESD should be validated in a prospective trial. In addition, we were not able to investigate intra‐procedural muscle injuries related to delayed perforation due to the retrospective nature of this study. Second, the health insurance system is quite different in Japan compared with other countries. Hospitalization may be restricted in some countries. The generalizability of our findings may be limited. Third, the expertise of the operators showed a wide variation, and one experienced endoscopist (Daiki Nemoto) performed quality control for all procedures in this series. This variation may affect short‐term outcomes.

In conclusion, no serious adverse events occurred in this series. Four days of hospitalization may be too long for the majority of patients after ESD. Clinical assessment on day 1 may be useful to predict the length of hospital stay for most patients. Based on the data in this study, patients treated with antithrombotic agents and those with rectal lesions are not good candidates for shortened hospital stay after colorectal ESD. Further clinical trials are warranted to evaluate these recommendations.

## CONFLICT OF INTEREST

The authors declare that they have no conflict of interest.

## FUNDING INFORMATION

None.

## Supporting information




**Table S1**. Modified Clavien‐Dindo Classification
**Table S2**. Features of patients with Clavien‐Dindo classification grade ≥ II adverse events
**Table S3**. Predictors of Clavien‐Dindo classification grade ≥ II adverse events
