# Supplementary material for: Short‐term outcomes of patients undergoing endoscopic submucosal dissection for colorectal lesions
Source: DEN Open. 2022 Jun 16;3(1):e136. doi: 10.1002/deo2.136 (PMC9307737; doi:10.1002/deo2.136)
Supplement: Supplementary file 1 — Table S1. Modified Clavien‐Dindo Classification Table S2. Features of patients with Clavien‐Dindo classification grade ≥ II adverse events Table S3. Predictors of Clavien‐Dindo classification grade ≥ II adverse events [file DEO2-3-e136-s001.docx]

| **Supplementary Table 1. Modified Clavien-Dindo Classification** | |
| --- | --- |
| Grade 0: | No adverse event |
| Grade I: | Any deviation from the normal postoperative course without the need for pharmacological treatment or  surgical, endoscopic, and radiological interventions Allowed therapeutic regimens include: drugs as antiemetics, antipyretics, analgesics, diuretics, electrolytes,  and physiotherapy. This grade also includes wound infections opened at the bedside |
| Grade II: | Requiring pharmacological treatment with drugs other than such allowed for grade I complications Blood transfusions and total parenteral nutrition are also included |
| Grade III: | Requiring surgical, endoscopic or radiological intervention |
| Grade IIIa: | Intervention not under general anesthesia |
| Grade IIIb: | Intervention under general anesthesia |
| Grade IV: | Life-threatening complication (including CNS complications)* requiring IC/ICU management |
| Grade IVa: | Single organ dysfunction (including dialysis) |
| Grade IVb: | Multiorgan dysfunction |
| Grade V: | Death of a patient |
| CNS, central nervous system; IC, intermediate care; ICU, intensive care unit. | |

| **Supplementary Table 2. Features of patients with Clavien-Dindo classification grade ≥ II adverse events** | | | | |
| --- | --- | --- | --- | --- |
| **Factor** | | ≤ Grade I (n=290) | Grade ≥ II (n=40) | p-value |
| Age (year), median (IQR) | | 72 (65-80) | 71 (64-81) | 0.63^§^ |
| Gender, n (%) | Male | 172 (59.3) | 26 (65.0) | 0.49^¶^ |
|  | Female | 118 (40.7) | 14 (35.0) |  |
| Antithrombotic agent use, n (%) | | 44 (15.2) | 6 (15.0) | 0.98^¶^ |
| Lesion location, n (%) | Proximal colon | 173 (59.7) | 25 (62.5) | 0.014^¶^ |
|  | Distal colon | 55 (19.0) | 1 (2.5) |  |
|  | Rectum | 62 (21.4) | 14 (35.0) |  |
| Lesion size (mm), median (IQR) | | 30 (22-39) | 35 (26-53) | 0.017^§^ |
| Morphology, n (%) | Flat | 142 (49.0) | 28 (70.0) | 0.024^¶^ |
|  | Polypoid | 148 (51.0) | 12 (30.0) |  |
| Histology, n (%) | Adenoma | 135 (46.6) | 21 (52.5) | 0.69^¶^ |
|  | Tis (superficial cancer) | 88 (30.3) | 10 (25.0) |  |
|  | T1a (submucosal invasion <1000µm) | 23 (7.9) | 4 (10.0) |  |
|  | T1b (submucosal invasion ≥1000µm) | 29 (10.0) | 5 (12.5) |  |
|  | SSL | 12 (4.1) | 0 (0) |  |
|  | Others^†^ | 3 (1.0) | 0 (0) |  |
| Cutting time (min), median (IQR) | | 55 (36-75) | 75^‡^ (63-112) | 0.00010^§^ |
| Intra-procedural perforation, n (%) | | 0 (0) | 10 (25.0) | <0.0001^¶^ |
| IQR: interquartile range; SSL: sessile serrated lesion | | | |  |
| Postoperative adverse events were evaluated using the modified Clavien-Dindo grade and categorized into two groups; ≤ grade I and ≥ grade II. | | | | |
| ^†^Others include 1 mucosal prolapse syndrome, 1 no residual tumor after previous endoscopic resection, and 1 dysplasia associated ulcerative colitis. | | | | |
| ^‡^One interrupted case is excluded. | |  |  |  |
| ^§^Mann-Whitney U test | |  |  |  |
| ^¶^chi-square test | |  |  |  |

| **Supplementary Table 3. Predictors of Clavien-Dindo classification grade ≥ II adverse events** | | | | | |
| --- | --- | --- | --- | --- | --- |
| **Factor** | | Univariate analysis^†^ | | Multivariate analysis^†^ | |
|  |  | OR (95% CI) | p-value | OR (95% CI) | p-value |
| Age (years) | <75 | 1 | 0.44 |  |  |
|  | ≥75 | 0.76 (0.39 - 1.5) |  |  |  |
| Gender | female | 1 | 0.51 |  |  |
|  | male | 1.3 (0.64 - 2.5) |  |  |  |
| Antithrombotic agent use | absent | 1 | 0.93 |  |  |
|  | present | 1.0 (0.42 - 2.6) |  |  |  |
| Lesion location | colon | 1 | 0.052 | 1 | 0.078 |
|  | rectum | 2.0 (0.99 - 4.0) |  | 2.1 (0.92 - 4.8) |  |
| Lesion size (mm) | <50 | 1 | 0.032 | 1 | 0.76 |
|  | ≥50 | 2.3 (1.1 - 4.8) |  | 1.2 (0.42 - 3.3) |  |
| Morphology | polypoid | 1 | 0.029 | 1 | 0.25 |
|  | flat | 0.46 (0.23 - 0.92) |  | 0.60 (0.26 - 1.4) |  |
| Histology | non-T1b | 1 | 0.52 |  |  |
|  | T1b | 1.4 (0.52 - 3.6) |  |  |  |
| Cutting time^‡^ (min) | <120 | 1 | <0.0001 | 1 | 0.032 |
|  | ≥120 | 4.2 (1.9 - 9.3) |  | 3.1 (1.1 - 8.9) |  |
| Intra-procedural perforation | absent | 1 | <0.0001 | 1 | <0.0001 |
|  | present | 200 (11 - 3500) |  | 170 (9.6 - 3100) |  |
| OR: odds ratio; CI: confidence interval | | |  |  |  |
| ^†^Logistic regression model with the Firth procedure. | | | | | |
| ^‡^One interrupted ESD is excluded from the analysis. | | | | | |
